# C-Type natriuretic peptide: a potential urinary biomarker for renal remodeling and fibrosis during aging

**DOI:** 10.1186/1471-2210-11-S1-P60

**Published:** 2011-08-01

**Authors:** Jeson S Sangaralingham, Denise M Heublein, Joseph P Grande, Alessandro Cataliotti, Andrew D Rule, Paul M McKie, Fernando L Martin, John C Burnett

**Affiliations:** 1 Cardiorenal Research Laboratory, Division of Cardiovascular Diseases, Mayo Clinic, Rochester, Minnesota 55905, USA; 2 Division of Nephrology and Hypertension, Department of Medicine, Mayo Clinic, Rochester, Minnesota 55905, USA; 3 Department of Laboratory Medicine and Pathology, Mayo Clinic, Rochester, Minnesota 55905, USA

## Background

Renal aging is characterized by structural changes in the kidney, including fibrosis, which are often accompanied by reduced glomerular filtration rate (GFR) and elevated urinary protein excretion (UPE) that likely contributes to increased risk of kidney failure, cardiovascular disease and mortality in the elderly. Importantly a recent study, using biopsy in healthy kidney donors across six decades of life, demonstrated the presence of preclinical age-related nephropathy which was otherwise undetectable using conventional clinical tests including GFR and UPE. Although, there are a number of biomarkers for renal disease and injury, there are few which target early renal remodeling prior to the onset of symptoms during natural aging. C-type natriuretic peptide (CNP) is highly expressed in renal cells, is potently anti-fibrotic, inhibits renal mesangial matrix accumulation and may play a role in podocyte-glomerular basement membrane (GBM) integrity. Since aging is associated with altered renal structure and function that may contribute to the development of kidney and heart failure, we sought to determine if CNP might serve as a urinary biomarker for global renal remodeling as a compensatory renal response to injury induced by aging.

## Objective

The objective of this study was to determine urinary CNP excretion in normal aging Fischer rats and its relationship to renal structure and function. We hypothesized that an increase in urinary CNP excretion will parallel increases in renal fibrosis and GBM thickening, which importantly, will precede an elevation in proteinuria in the aging kidney.

## Methods

Studies were performed in 2, 11 and 20 month old male Fischer rats (equivalent to human aging from childhood to the 6th decade of life; n=8-10/group). Blood pressure (BP) UPE, GFR, urinary CNP was measured. Kidneys harvested for histologic and electron microscopic analysis. Mean±SE, +P<0.05 vs 2 months, † P<0.05 vs 11 months.

## Results

Aging from 2 to 11 to 20 months was associated significant ang progressive renal cortial (1±0.3 vs 8±2* vs 17±1* † %) and medullary (4±0.8 vs 11±1* vs 17±1* † %) fibrosis, as well as GBM thickening (120±13 vs 305±12* vs 535±20*† nm). Further, electron microscopy revealed progressive mesangial matrix accumulation at 11 and 20 months, along with focal effacement of visceral epithelial cell foot processes of the podocyte at 20 months. Importantly, urinary CNP excretion significantly increased at 11 months, which was associated with early non-pathological renal fibrosis and glomerular remodeling as described, and remained elevated at 20 months (64±4 vs 110±vs 103±pg/day). BP and UPE were mildly but significantly elevated at 20 months (90±1 vs 90±2 vs 100±3*† mmHG and 8.1±0.6 vs 8.2±0.4 vs 20±3*† mg/day, respectively). Further, GFR decreased at 11 months and was sustained at 20 months (3.52±0.29 vs 2.55±0.37 vs 2.83±0.30 ml/min/kg).

## Conclusion

We report for the first time that a significant increase in urinary CNP excretion during aging is strongly association with global renal fibrosis and GBM thickening, which occurred prior to the onset of significant proteinuria or BP elevation. Further studies are warranted to explore the idea that urinary CNP may serve as a sensitive urinary biomarker to detect the presence of preclinical renal structural remodeling and/or impairment, particularly during aging, to facilitate early diagnosis and intervention to reduce the burden of chronic kidney disease.

